# Alveolar Echinococcosis of the Parotid Gland—An Ultra Rare Location Reported from Western Europe

**DOI:** 10.3390/pathogens10040426

**Published:** 2021-04-03

**Authors:** Tim Koppen, Thomas F. E. Barth, Klaus W. Eichhorn, Jennis Gabrielpillai, Ralph Kader, Friedrich Bootz, Thorsten Send

**Affiliations:** 1 Department of Otorhinolaryngology, Head and Neck Surgery, University Medical Center Bonn, Venusberg-Campus 1, 53127 Bonn, Germany; klaus.eichhorn@ukbonn.de (K.W.E.); Jennis.Gabrielpillai@ukbonn.de (J.G.); Friedrich.Bootz@ukbonn.de (F.B.); thorsten.Send@ukbonn.de (T.S.); 2 Institute of Pathology, University of Ulm, Albert-Einstein Allee 11, 89081 Ulm, Germany; thomas.barth@uniklinik-ulm.de; 3 Institute of Radiology and Nuclear Medicine at the Kaiser-Passage in Bonn, Martinsplatz 2a, 53113 Bonn, Germany; ralph_kader@web.de

**Keywords:** *Alveolar echinococcosis*, parotid gland, abscess, case report

## Abstract

(1) Background: *Alveolar echinococcosis* (AE) is restricted to the northern hemisphere with high endemic regions in Central Europe, North and Central Asia as well as Western China. The larval stage of *Echinococcus multilocularis (E. multilocularis)* causes AE with tumor-like growth. Humans are accidental hosts. This report is on the first case of AE becoming clinically manifested in the parotic gland. (2) Case presentation: A 52-year-old male patient presented with progressive and painful swelling of the right parotid gland persisting for one year. We performed a partial parotidectomy. The histological examination and immunohistological staining revealed larval stage of *E. multilocularis*. (3) Conclusion: *E. multilocularis* is known to infect animals and humans coincidentally, and leads to AE. It is one of the most life-threatening zoonoses in Europe. It typically manifests in the liver (50–77%), with further spreading to other organs being a rare phenomenon. Echinococcosis should be considered in the differential diagnosis of lesions of the parotid gland in endemic areas, but AE has not been described so far in the parotid gland as the sole manifestation and, therefore, impedes the correct diagnosis. A complete resection should be the aim, however, preservation of the facial nerve and adjuvant albendazole therapy is mandatory.

## 1. Introduction

Infections of humans with the tapeworm *Echinococcus spp.* are a global burden [1,2]. The two main types of echinococcosis are *cystic echinococcosis* (CE) caused by the *Echinococcus granulosus* (EG) species complex and *alveolar echinococcosis* (AE) [3]. AE is restricted to the northern hemisphere, with high endemic regions in Central Europe, North and Central Asia, and Western China [4]. Humans are accidental intermediate hosts of AE. The larval stage of *Echinococcus multilocularis (E. multilocularis*) causes AE with tumor-like growth due to ingestion of eggs, released with the feces of infected animals of the canid family, including foxes. AE manifests mainly in the liver and rarely in other organs such as the lungs, heart or spleen [4]. Development of AE can take years in humans, but leads to death if this zoonotic disease remains untreated (up to 90%) [4,5]. The parotid gland is an extremely rare location—even for CE—and in this paper, we report on the first case of AE becoming clinically manifested in the parotic gland. This report is about diagnostics and therapy of a patient with an ultra rare manifestation of AE in the parotid gland who was treated in the otorhinolaryngology department of the University Medical Center Bonn (Germany).

## 2. Results

**Figure 1:**
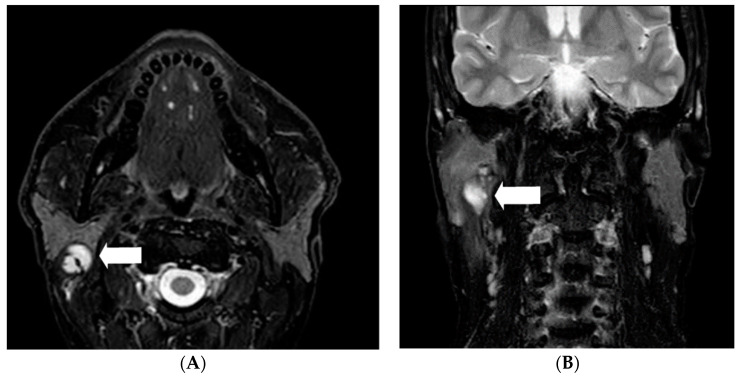
MRI of head and neck, 1.5 tesla magnet. T2 fatsat imaging in axial (**A**) and coronal (**B**) orientation. Cystic lobulated tumor in the medial lobe of parotid gland adjacent to the facial nerve (arrow).

**Figure 2:**
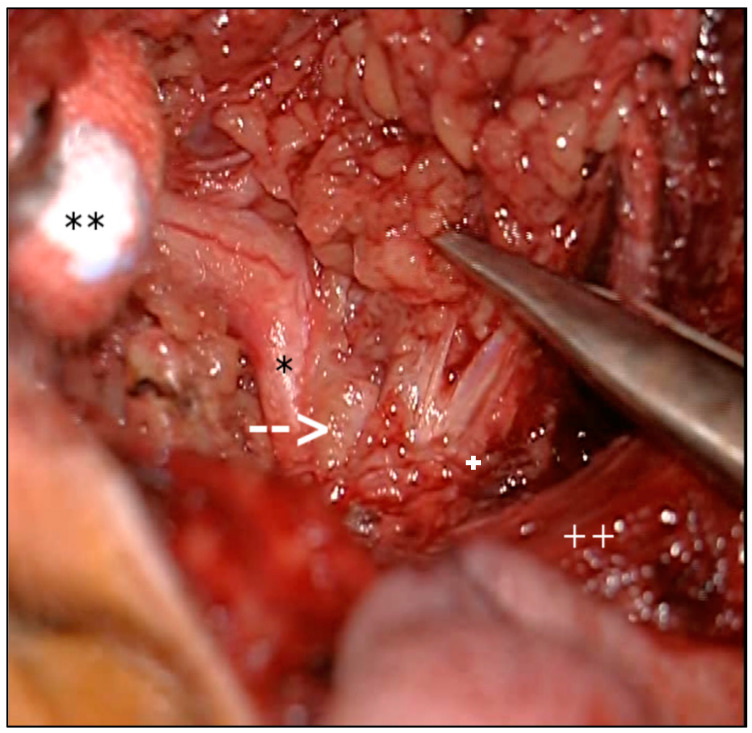
Imaging during surgery reveals a cystic tumor surrounding the parotid gland (facial nerve (*); rami zygomaticus and frontalis (**); cystic tumor (arrow); ramus marginalis mandibulae (+); sternocleidomastoid muscle (++)).

A 52-year-old male patient presented with progressive and painful swelling of the right parotid gland: a state he had been in for one year, beginning in March 2018. He did not show a facial nerve palsy. There was no history of any medication, known allergies or chronic or rheumatic diseases. The patient was born in Germany, lived within West European standards and had been employed as a physician at a university hospital at the time when he was first diagnosed. He lived in a town with 25,000 citizens in the Rhein-Sieg district, in the state of North Rhine-Westphalia, Germany, situated approximately 15 km south-west of the city Bonn. The patient avoided journeys to high risk areas. He had no pet animals and no specific leisure activities such as hunting. There are some vineyards in this area which he passed by daily on his way to work. The initial differential diagnosis after magnetic resonance imaging (MRI) was an abscess in the right medial parotid gland with a diameter of 2.7 × 1.6 × 1.6 cm and involvement of the ipsilateral sternocleidomastoid muscle (Figure 1). Initial antibiotic therapy with cefuroxime and metronidazole did not elicit an effect. Therefore, we performed a partial parotidectomy. The situs showed a strong inflammatory reaction including a cystic structured tumor reaching the main trunk of the facial nerve, surrounding the onset of the sternocleidomastoid muscle. The lesion was removed (Figure 2) and was subject of histopathological analysis. A full-body MRI-scan revealed no further manifestations, except a small liver cyst. It measured 1.0 cm and had been known about for more than ten years. Medical records revealed a negative serum screening test for AE and CE (anti-Echinococcus-ELISA (Enzyme-linked Immunosorbent Assay) IgG (immunoglobulin G), Euroimmun, Lübeck, Germany) and a negative qualitative *E. multilocularis* Western blot IgG (LDBIO Diagnostics, Lyon, France) taken in 2016.

**Figure 3:**
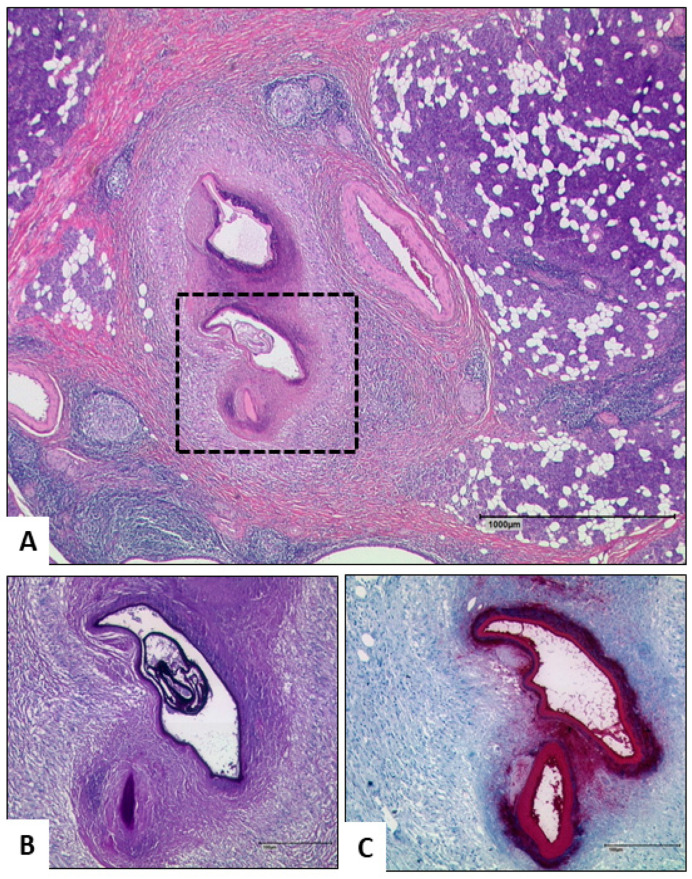
(**A**,**B**) Histology of the parotic gland shows a pseudocystic lesion with strong chronic and necrotic inflammation in the parenchym with induction of fibrosis. Immunohistochemical staining with the monoclonal antibody Em2G11 (**C**) revealed a slender structure as residuum of the larval stage of *E. multilocularis.*

The histological examination showed chronic granulating and necrotizing inflammation. A Periodic acid–Schiff reaction staining (PAS) showed deeply blue stained slender structures surrounded by necrotic material. Further differentiation was achieved by immunohistological staining with the monoclonal antibody Em2G11 (mAbEm2G11), specific for a glycoprotein (Em2) of the laminated layer of the *E. multilocularis* larval stage in formalin fixed paraffin-embedded (FFPE) human tissue blocks (Figure 3) [6]. Polymerase chain reaction (PCR) for the detection of tapeworm 12S rDNA sequences was additionally performed from FFPE tissue to add molecular diagnostics but could not confirm an infection with cestodes [7].

We started a systemic therapy with albendazole (400 mg 1-0-1 per os) over a period of two years minimum. The initial MRI-scan of the body revealed no other organ manifestations. A follow-up MRI revealed no relapse. We performed a PubMed search for the keywords “echinococcus + parotid gland (± hydatid cyst; ± alveolar echinococcosis)” (Table 1) and found no case of *E. multilocularis* manifestation described in the parotid gland.

## 3. Discussion

*E. multilocularis* is a parasite of the cestode group. Infection with the larval stage leads to AE. *E. multilocularis* is known to infect animals and humans coincidentally. AE is one of the most life-threatening zoonoses in Europe [8,9]. Canidae and cats are definitive hosts, while rodents serve as intermediate hosts harboring the larval stage of *E. multilocularis*. The life cycle is completed as infected animals, such as mice, are prey to the definitive hosts, such as the red fox. Humans are accidental intermediate hosts. After the ingestion of eggs, the oncospheres transform to the larval stage called a metacestode, which slowly grows to form a tumor-like parasitic tissue mass, predominantly in the liver.

AE may manifest in any organ, but typically in the liver (50–77%), and rarely in the spleen or thoracic organs such as the lungs and heart since the portal vein system works as a filter. Hence, further spreading to other organs is rare [8,10]. In contrast to mainly liver restricted AE, CE—caused by *E. granulosus*—has occasionally been described as a rare manifestation in the region of the head and neck. Research of the literature resulted in us determining that only 1% of humans with CE develop cysts in head and neck region [11]. At least 29.6 % of oromaxillofacial manifestations are located in the parotid gland. A total of 17.6% of the cases showed other organs to be involved [12]. To the best of our knowledge, AE has not been described in this area yet (Table 1) [11,12].

The most important information in terms of the location and adjacent structures is obtained by ultrasound, computer tomography (CT)-scan or MRI-scan. Serological findings, such as ELISA and indirect haemagglutinations are extremely useful to confirm the diagnosis, despite possible false-positive results (13.9%), especially in extrahepatic disease [5]. Since earlier serological tests performed in 2016 of our patient were negative, seroconversion must have occurred during the last two years. The immunohistological staining with the monoclonal antibody Em2G11 corroborated AE of the right parotid gland caused by the larval stage of *E. multilocularis* (Figure 3), but Polymerase chain reaction (PCR) could not. The applied PCR-method detects tapeworm 12S rDNA sequences of as little as 50 parasite cells per specimen, in immunohistostained tissue areas after micro-dissection [7]. However, the authors describe that the method could not detect *E. multilocularis* DNA in 11 of 15 samples in tissue with mAbEm2G11-positive small particles of *E. multilocularis* (spems) and showed negative results due to spems contained on the surface material when the parasite probably did not contain parasite cells. Furthermore, the diagnostic sensitivity of material containing only spems was higher using immunhistostaining. We analyzed available FFPE tissue. After being stored over several years, either missing parasitic DNA and viable cells of the parasite, or sample preparation caused by DNA fragmentation could represent the reasons for the negative PCR results. The combination of immunohistochemical staining (Em2G11) and PCR (12S rDNA) are reliable means to unequivocally detect infection. Therefore, these results do not contradict prior findings.

We decided to perform surgical therapy with almost complete removal of the affected tissue, leaving some tissue of the lesion close to the facial nerve and started postoperative antihelmintic drug therapy. In the literature, the combination of surgery and medication is only performed in about 23% of CE, whereas an exclusively surgical treatment has been performed in 66% in head and neck area cases [12]. Differential diagnoses include other local tumor diseases such as congenital cysts, dermoid or epidermoid cysts, branchial cleft cysts or cystic hygromas. Some may be associated with human immunodeficiency virus (HIV) infection or autoimmune diseases, such as Sjögren syndrome, systemic lupus erythematodes, polyarteritis nodosa, polymyositis or scleroderma [9].

## 4. Conclusions

Echinococcosis should be considered in the differential diagnosis of lesions of the parotid gland in endemic areas—especially for CE—but it has not been described so far for AE in the parotid gland as the sole manifestation.

The manifestation of AE limited to the parotid gland is extremely uncommon and, therefore, impedes correct diagnosis. Complete resection should be the aim, however, preservation of the facial nerve and adjuvant albendazole therapy is mandatory.

## Figures and Tables

**Table 1:** Case reports of *Echinococcus* findings in the parotid gland listed in Medline (searched keywords: “echinococcus + parotid gland (±hydatid cyst ± alveolar echinococcosis”)), from 1980.

Author	Cases	*E. multilocularis* (EM) *E. granulosus* (EG)	Additional Organs Involved	Region	Age in Years
Erdur O et al., 2018	1	EG	No	Turkey	26
Kara T et al., 2017	1	EG	No	Turkey	54
Arora V K et al., 2016	1	EG	No	India	13
Diwan R et al., 2015	1	EG	No	India	35
Du Plessis J et al., 2012	1	EG	No	South Africa	20
Zouita B et al., 2011	1	EG	No	Morocco	10
Darabi M et al., 2008	1	EG	No	Iran	23
Safiolias M et al., 2007	1	EG	No	Greece	40
Karahatay S et al., 2006	1	EG	No	Turkey	-
Oudidi A et al., 2006	6	assumed EG	No	Morocco	-
Divisi D et al., 2006	1	EG	Lung	Italy	81
Akhan O et al., 2002	1	assumed EG	-	Turkey	-
Develoux M et al., 1985	1	assumed EG	No	Niger	-
Kalovidouris A et al., 1985	1	assumed EG	Fossa infratemporalis	Greece	-
Saxena S K et al., 1983	1	EG	No	Lebanon	45
***REVIEWS/SERIES***					
El Bousaadani A et al., 2016	6	assumed EG	-	Morocco	Av. 35
Just B A et al., 2014	32	assumed EG	-	-	Av. 22
Belcadhi M et al., 2011	2	-	-	-	Av. 27
Bellil S et al., 2009	1	-	-	Tunisia	Av. 38.7
Cooney R et al., 2004	3	assumed EG	-	Kenya	Av. 27

Average age = Av., no information given = -.

## Data Availability

The datasets used and/or analyzed in the course of the current study are available from the corresponding author on reasonable request.

## Abbreviations

AE	Alveolar echinococcosis
CE	Cystic Echinococcosis
*E. multilocularis*	*Echinococcus multilocularis*
ELISA	Enzyme-linked Immunosorbent Assay
FFPE	Formalin fixed paraffin-embedded
IgG	Immunoglobulin G
MRI	Magnetic resonance imaging
PAS	Periodic acid-Schiff reaction staining
PCR	Polymerase chain reaction
Spems	Small particles of *E. multilocularis*
